# Safety and Efficacy of Concurrent Atezolizumab/Bevacizumab or Nivolumab Combination Therapy with Yttrium-90 Radioembolization of Advanced Unresectable Hepatocellular Carcinoma

**DOI:** 10.3390/curroncol30120734

**Published:** 2023-11-25

**Authors:** Alexander Villalobos, Howard Hussein Dabbous, Olivia Little, Olumide Babajide Gbolahan, Mehmet Akce, Meghan Allegra Lilly, Zachary Bercu, Nima Kokabi

**Affiliations:** 1 Department of Radiology, University of North Carolina at Chapel Hill, Alexander Villalobos 101 Manning Drive, Chapel Hill, NC 27514, USA; nima_kokabi@med.unc.edu; 2 Department of Radiology and Imaging Sciences, Emory University School of Medicine, Atlanta, GA 30322, USA; hdabbous@dmc.org (H.H.D.); meghan.lilly@emory.edu (M.A.L.); zachary.louis.bercu@emory.edu (Z.B.); 3 Department of Radiology and Imaging Sciences, Mercer University School of Medicine, Savannah, GA 31404, USA; olivia.lekuang.little@live.mercer.edu; 4 Department of Hematology and Medical Oncology, Emory University School of Medicine, Atlanta, GA 30322, USA; ogbolah@emory.edu; 5 Department of Hematology and Medical Oncology, University of Alabama School of Medicine, Birmingham, AL 35294, USA; makce@uabmc.edu

**Keywords:** Y90 radioembolization, SIRT, nivolumab, atezolizumab, bevacizumab, anti-PD-L1, immune checkpoint inhibitors, hepatocellular carcinoma

## Abstract

To evaluate the safety and efficacy of combining yttrium-90 radioembolization (Y90-RE) with immune checkpoint inhibitor therapy, consecutive advanced unresectable hepatocellular carcinoma (HCC) patients treated between 2016 and 2022 with atezolizumab/bevacizumab or nivolumab within three-months pre- and post-Y90-RE were retrospectively evaluated. Tumor response and treatment-related clinical/laboratory adverse events (AE) were assessed at 1 and 6 months, as well as differences in clinical and laboratory variables and median overall survival (OS) from initial treatment (whether it was Y90-RE or systemic therapy) between the two cohorts. A total of 19 patients (10 atezolizumab/bevacizumab; 9 nivolumab), comprising 84% males with median age 69 years, met the inclusion criteria. Compared to the atezolizumab/bevacizumab group, there were less males (100% vs. 67%; *p* = 0.02) and more ECOG ≥ 2 patients in the nivolumab group (0% vs. 33%; *p* = 0.02). Baseline characteristics or incidence of 6-month post-treatment any-grade AE (60% vs. 56%; *p* = 0.7), grade ≥ 3 AE (0% vs. 11%; *p* = 0.3), objective response (58% total, 60% vs. 56%; *p* = 0.7), and complete response (16% total; 10% vs. 22%; *p* = 0.8) were similar between the atezolizumab/bevacizumab and the nivolumab cohorts. Median OS was 12.9 months for the whole cohort, 16.4 months for nivolumab, and 10.7 months for atezolizumab/bevacizumab. Among patients with advanced unresectable HCC, the utilization of Y90-RE concurrently or within 90 days of nivolumab or atezolizumab/bevacizumab immunotherapy, appears to be well-tolerated and with a low incidence of severe AE.

## 1. Introduction

Hepatocellular carcinoma (HCC) is the most common type of liver cancer, the seventh most common cancer in the world, and is the fourth leading cause of cancer mortality worldwide [1]. Out of the hundreds of thousands of newly diagnosed HCCs annually, more than 50% of these patients will be diagnosed at an advanced stage of either Barcelona Clinic Liver Cancer (BCLC) stage C or D and will be considered unsuitable for local therapy or surgery at most treating centers [2]. The median overall survival (OS) in untreated HCC decreases drastically as the cancer stage worsens, with BCLC stage A, B, C, and D having respective median OS of 25, 10, 7, and 6 months [3]. The current standard of care for advanced unresectable HCC is systemic therapy. Yttrium-90 radioembolization (Y90-RE) is an alternative in advanced HCC patients with contraindications to systemic therapy with liver-confined disease and favorable liver function [4]. The first systematic therapy approved for use in advanced unresectable HCC was sorafenib, a multiple kinase inhibitor, in 2007, and remained the only drug FDA-approved until 2016 [5]. The original study for sorafenib demonstrated a median OS of 10.7 compared to 7.9 months in the placebo group [6]. Since then, however, multiple new drugs (tyrosine kinase inhibitors (TKIs), anti-programmed death-1 (PD-1) (e.g., nivolumab), and anti-PD-L1 therapies (e.g., atezolizumab)), and their combinations, have been approved for first- and second-line therapy for advanced HCC [7,8,9,10,11,12,13]. Atezolizumab (anti PD-L1 therapy) and bevacizumab (anti-vascular endothelial growth factor (VEGF)) was studied in the IMBrave-150 trial and was shown to be superior to sorafenib therapy in the treatment of advanced unresectable HCC [7]. This study led to the approval of atezolizumab/bevacizumab dual-agent therapy in the frontline treatment for advanced unresectable HCC [14]. However, not all patients can receive bevacizumab as it increases the risk of bleeding (which can be particularly concerning in cirrhotic patients with varices) or vessel friability (which can limit the utilization of angiographic procedures). More recently, durvalumab and tremelimumab combination therapy was approved for the front-line therapy of advanced HCC based on the HIMALAYA trial [8]. Nevertheless, there remains a need for investigations aimed to identify the most effective treatment for HCC, including with the combination of currently approved systemic therapy options and locoregional therapies (e.g., Y90-RE). 

As multicenter, open-label, randomized, controlled, phase 3 trials, the SARAH and SIRveNIB trials previously compared Y90-RE to sorafenib [15,16]. However, both trials yielded no difference in median OS, with the SARAH and SIRveNIB studies demonstrating 8.0 vs. 9.9 months and 8.8 vs. 10.0 months for Y90-RE vs. sorafenib cohorts, respectively. A growing interest towards combining systemic therapy with Y90-RE was then explored, with the multicenter, open-label, randomized, controlled, phase 2 SORAMIC trial being one of the first major trials to explore this sort of combination therapy by evaluating the efficacy and safety of sorafenib with Y90-RE combined therapy for advanced HCC [17]. Nevertheless, this study also reported no significant increase in OS for patients receiving both Y90-RE and sorafenib. Since then, there have been relatively few studies evaluating the combination of Y90-RE and systemic therapies in the treatment of advanced unresectable HCC. 

Because HCC progression is, in part, attributed to the evasion of immune surveillance [18], immune checkpoint inhibitors have shown promise in their ability to ameliorate HCC’s anti-tumor immunity by inhibiting the activation of immune checkpoints. While early trials (such as CheckMate459 for nivolumab) evaluating the efficacy of immuno-monotherapy for the treatment of HCC failed to show an increase in overall survival compared to that of standard of care (sorafenib), these trials did demonstrate the capability for immunotherapy agents to be better tolerated than other systemic therapy options [18]. This has led to the significant interest in the exploration of these novel immune modulating agents for their use in the treatment of HCC, including as a combination therapy with locoregional therapies, such as Y90-RE [19]. The hypothesis for the synergistic effect of combining Y90-RE with systemic immunotherapy is that Y90-RE can stimulate an inflammatory response that releases antigenic tumor loads that can stimulate the immune system to recognize and attack the cancer cells [19]. In short, the utilization of Y90-RE can augment an immunotherapy’s ability to suppress HCC’s anti-immune-recognition capabilities, thereby providing, in theory, a more robust tumor response.

There remains, to date, a paucity of studies evaluating the use of immunotherapies in combination with Y90-RE for the treatment of advanced unresectable HCC. Our study aims to address this knowledge gap by investigating the efficacy and safety of performing combination therapy with Y90-RE and immune checkpoint inhibitors, namely, atezolizumab/bevacizumab or nivolumab. 

## 2. Materials and Methods 

### 2.1. Study Design and Population 

A single tertiary-center retrospective study was performed, with institutional review board approval and HIPAA compliance, at an academic transplant institution. The inclusion criteria included ≥18 years old adult patients with advanced unresectable HCC who received systemic checkpoint inhibitor therapy (either atezolizumab/bevacizumab combination therapy, or nivolumab single-agent therapy) within three months pre- and post-Y90-RE. Additionally, captured patients must have had complete pre-, 1-month-post-, and 6-month-post-Y90-RE clinical and imaging follow-ups. The exclusion criteria included alternative diagnosis on liver tissue sampling (e.g., mixed HCC–cholangiocarcinoma or cholangiocarcinoma) or any prior Y90-RE therapies to the targeted lesion. Due to the FDA approval timeline for HCC immunotherapy, only patients who underwent Y90-RE at the study institution between 2016 and 2022 were screened. Consecutive patients with locally advanced HCC who were deemed to benefit from the combination of systemic therapy and liver-directed therapy with Y90-RE by a multidisciplinary institutional tumor board were included. Of note, patients at the study’s institution received atezolizumab/bevacizumab combination therapy as part of their systemic therapy if they did not have gastroesophageal variceal disease or if they had gastroesophageal variceal disease that was amenable to banding. If patients had significant gastroesophageal variceal disease that was not amenable to banding, patients at the study’s institution received nivolumab instead. For those patients who were initiated on an atezolizumab/bevacizumab regimen prior to Y90-RE, bevacizumab was held for four weeks prior to Y90-RE and restarted one week after. Atezolizumab was held for 1 week post-Y90 before recommencement of both atezolizumab/bevacizumab agents together. For patients receiving nivolumab before Y90-RE, nivolumab was only held for 1 week after Y90-RE before recommencement. In patients who were initiated on either form of systemic therapy after Y90-RE, there was no dose modification or holding of systemic therapy. 

### 2.2. Clinical Information and Outcomes 

The baseline characteristics captured included age, gender, ethnicity, and Eastern Cooperative Oncology Group (ECOG) performance scores. Clinical and laboratory history were also obtained, including history of any prior local therapy to targeted lesions, prior systemic therapy, etiologies of liver disease, Barcelona Clinic Liver Cancer (BCLC) stage, Child–Pugh (CP) class, model end-stage liver disease (MELD) score, albumin-bilirubin (ALBI) scores, albumin-to-alkaline phosphatase ratio (AAPR), and baseline serum apha-fetoprotein (AFP).

Clinical and laboratory adverse events (AE) were graded utilizing the Common Terminology Criteria for Adverse Events v5.0 (CTCAE) grading system [20]. The evaluated clinical AEs consisted of encephalopathy, ascites, fatigue, abdominal pain, nausea, vomiting, anorexia, constipation, and fever. The evaluated laboratory AEs included serum white blood cell count (WBC), neutrophils, lymphocytes, creatinine, sodium, albumin, total bilirubin, aspartate aminotransferase, alanine aminotransferase, alkaline phosphatase, and international normalized ratio (INR). Overall survival (OS) was calculated from the date of the protocol start (defined as the initial day of treatment, whether it was Y90-RE or systemic therapy) to the patient’s death or last known follow-up.

### 2.3. Imaging Information

The baseline HCC imaging information and characteristics were taken from the pre-treatment imaging 30 days prior to the initiation of therapy, while the post-treatment imaging data were captured from the 1-month and 6-months imaging follow-ups. Additionally, the baseline image data included the type of Y90 microsphere utilized (resin-based vs. glass-based), if the patient underwent Y90-radioembolization segmentectomy (Y90-RS) or lobar therapy, evidence of cirrhosis, targeted tumor size, macrovascular invasion, extrahepatic spread of HCC, and the presence of other non-targeted HCC tumors within the liver. The imaging was acquired utilizing a dynamic contrast-enhanced (DCE) liver protocol in either magnetic resonance images (MRI) or computed tomography (CT) images. For CT imaging, a multi-detector-row helical CT scanner (Light Speed VCT; GE Medical Systems, Milwaukee, WI, USA) was utilized. For MRI, a 1.5/3-T MR scanner (Siemens, Erlangen, Germany) or a 1.5-T scanner (GE Medical Systems) was used to acquire the MR images. Arterial, venous, and delayed phase images were acquired at 20 s, 70 s, and 180 s, respectively. The imaging tumor response to the Y90-RE was retrospectively evaluated by an abdominal-fellowship-trained radiologist using the modified Response Evaluation Criteria in Solid Tumors (mRECIST) criteria for HCC treatment [21], and the imaging outcomes were reported as complete response, partial response, stable disease, or progressive disease. For analysis purposes, an objective response was defined as a complete response or a partial response, while disease control was defined as an objective response or stable disease. 

### 2.4. Radioembolization Procedure 

Prior to the Y90-RE procedure, a shunt study was first conducted to delineate and assess the mesenteric, extrahepatic, and intrahepatic vasculature as previously described in the previous literature [22]. During this planning study, arteries that were angiographically found to be perfusing the targeted tumors were selected by the interventional radiologist prior to the injection of technetium-99m macroaggregated albumin (Tc-99m MAA) for confirmation of complete tumor coverage. Following delivery of the Tc-99m MAA, planar and single-photon emission computed tomography CT (SPECT/CT) was performed to calculate the lung shunt fraction and assess for any extrahepatic activity.

The prescribed Y90 activity for Y90-RS using both glass and resin microspheres was calculated using the medical internal radiation dose (MIRD) model [23]. Specifically, all resin microspheres activity was calculated utilizing the 3-day pre-calibration Y90 activity needed to reach a targeted segment dose of 150 gray (Gy) [24]. On the other hand, most of the glass-based Y90-RS cases were planned with a target dose of 190 Gy [25] to the segment, while a few were planned 120 Gy to the lobe but with dose delivery to the segment (at the discretion of the interventional radiologist and the planning nuclear medicine physician). For lobar treatments, the glass microsphere cases were planned with targeted 120 Gy to the perfused lobe of the liver using MIRD. For the resin microspheres lobar therapies, a partition model was used with the goal of >120 Gy to the targeted tumor while ensuring the dose to the whole non-tumoral liver volume remained below 40 Gy [26].

After determining the Y90 activity, the calculated activity was administered in the exact same arterial location as where the Tc-99m MAA delivery occurred during the planning shunt study. Immediately post-Y90-RE, patients underwent Y90 Bremsstrahlung (Brem) SPECT/CT to verify the administration of the prescribed activity to the targeted liver tumor(s).

### 2.5. Statistical Analysis 

Continuous variables were compared using a Student’s *t*-test when suitable, and categorical variables were compared using the chi-square test. Statistical significance was set at <0.05. The median OS from initial treatment (whether it was Y90-RE or systemic therapy) was calculated using the Kaplan–Meier estimation. JMP statistical software (JMP Pro, Version 15. SAS Institute Inc., Cary, NC, USA) was utilized to perform all the statistical analyses in this study.

## 3. Results 

### 3.1. Patient Demographics 

A total of 19 patients with advanced unresectable HCC were retrospectively identified and found to have undergone Y90-RE within three months of systemic checkpoint inhibitor therapy. Most patients were Caucasian (58%) and male (84%) with a median age of 69 years. Fifty-three percent had hepatitis C and 79% had liver cirrhosis. Overall, ten patients (53%) received atezolizumab as systemic immunotherapy, and nine (47%) received nivolumab (Table 1). The most common baseline clinical characteristic for the cohort was an ECOG Grade 1 (47%), ALBI Grade 2 (53%), CP Class A (89%), BCLC Stage C (84%), median MELD-Na score of 10, a median AAPR score of 0.37, and a median lung shunt fraction of 5%. Most of the cohort had an AFP ≤ 400 ng/mL (63%), no prior local therapy to the targeted lesion (74%), and no prior systemic therapy (84%). Most patients underwent Y90-RE lobar treatment (63%) with glass spheres (58%) to a tumor having a median diameter of 3.5 cm. 

Overall, with the exception for the nivolumab group having more female patients and higher ECOG scores than the atezolizumab/bevacizumab group, the baseline characteristics between the nivolumab and the atezolizumab/bevacizumab groups were similar (Table 1). There was no significant difference in the baseline imaging characteristics between the two cohorts (Table 2). 

### 3.2. Treatment Outcome and Toxicity 

The rates of objective response and disease control at 1 and 6 months were 58% and 63% for the overall cohort, respectively. No significant difference in the tumor response outcomes, as evaluated by mRECIST, was noted at 1 month (Supplementary Materials, Table SA) or at 6 months (Table 3) between the two cohorts. 

The incidences of any grade clinical AE or any ≥3 grade clinical AE were 58% and 5% for the whole cohort at 6 months, respectively. The incidences of any grade laboratory AE or any ≥3 grade laboratory AE were 84% and 37% for the whole cohort at 6 months, respectively. At 6 months, the most common incidence of any clinical AE was fatigue (47%), while the most common laboratory AE incidence involved lymphocytes (84%). Both systemic treatment regimens combined with Y90-RE had similar treatment profiles at 1 month (Supplementary Materials, Table SB) and at 6 months (Table 4), with the nivolumab group demonstrating a slightly increased amount of laboratory AE occurrence involving albumin than in the atezolizumab/bevacizumab group at 1 month. There was no Grade 4 clinical/laboratory AE for either cohort.

The OS for the overall cohort had a median time of 12.9 months. The OS for the nivolumab and atezolizumab/bevacizumab groups were 16.4 and 10.7 months, respectively. The median follow-up time for the entire cohort was 19 months as of June 2023. 

## 4. Discussion 

In this retrospective study, the utilization of Y90-RE within three months of systemic checkpoint inhibitor therapy was found to be safe and well-tolerated among patients with advanced unresectable HCC. While there are many ongoing clinical trials that have yet to be concluded ([27,28], NCT05809869, NCT03889093, NCT05701488, NCT04605731), there are few published studies that have evaluated the efficacy and safety of Y90-RE combined with an immune checkpoint inhibitor. In a single-center retrospective study that evaluated the impact of Y90-RE on 21 HCC patients who received Y90-RE 60 days before or concurrently with nivolumab, it was found that the median OS after Y90-RE was 12.0 months, and roughly 14.8 months from nivolumab initiation (i.e., the start of the treatment protocol) [29]. They also found that severe AEs (i.e., Grade 3 and 4 AEs) occurred in 5% and 13% of patients by months 1 and 3, respectively. In another single-center retrospective study of 26 patients who underwent Y90-RE concurrently or before/after 90 days of nivolumab or nivolumab/ipilimumab therapy, the median OS from first immunotherapy was 17.2 months, and 16.5 months from first Y90-RE for the overall study cohort [30]. Severe AEs were noted in 20% of their patient cohort, of which the authors suggested that some of the observed AEs may have been a consequence of the patient’s tumor biology/progression, and not necessarily of the combined Y90-RE plus immunotherapy regimens, causing liver injury. A phase 2, single-arm, multicenter clinical trial (NCT03380130) that evaluated the safety and efficacy of starting nivolumab 3 weeks after Y90-RE for HCC found the median OS (from the time of Y90-RE) to be 20.9 months, with an overall severe AE incidence of 26% [31]. It is worth noting, however, that most of the patients in that prospective study were of BCLC stage B and ECOG 0, which is a different population than the one characterized in this study and in the two previously mentioned retrospective studies. In another phase 2 clinical trial (NCT03033446) that evaluated the safety and efficacy of starting nivolumab 3 weeks after Y90-RE for HCC in an Asian population, it was found that the median OS (from time of Y90-RE) for those patients without extrahepatic spread was 20.2 months, while the incidence of severe AE for the overall cohort with advanced HCCs was 14% [32]. Lastly, in a phase 1, multi-center clinical trial (NCT03099564) that evaluated the safety and efficacy of performing Y90-RE on Y90-RE naïve “poor prognosis HCC” patients (multifocal disease, branch portal vein thrombosis, and/or diffuse liver tumor burden without extrahepatic metastasis) one week after pembrolizumab (anti-PD-1) dosing, the median OS was found to be 22 months [33]. To date, there are no published studies (but some soon to be published [27,28]) directly evaluating the efficacy and safety of combined Y90-RE with atezolizumab/bevacizumab therapy for advanced unresectable HCC. 

In this study, the median OS of the overall cohort was 12.9, with the nivolumab and atezolizumab/bevacizumab group demonstrating a median OS of 16.4 months and 10.7 months, respectively. While the median OS for this study’s nivolumab group fits nicely within the results of similar published studies, this study’s median OS for the atezolizumab/bevacizumab cohort was found to be less than that published in the IMbrave150 trial [7]. The incidence of severe AEs in this study was minimal to none (especially if one were to exclude lymphocyte AEs, which is a metric not frequently evaluated in the above-mentioned studies), a finding comparable to the above-mentioned retrospective and prospective study results for nivolumab. Regarding the atezolizumab/bevacizumab cohort, the incidence of severe AEs in this study is arguably comparable to the similarly reported severe AE in the IMbrave150 trial, reaffirming the safety of combining atezolizumab/bevacizumab with Y90-RE.

The intent of this retrospective study was to report the efficacy and patient tolerability of the combined Y90-RE plus systemic immunotherapy with a single-agent nivolumab or atezolizumab/bevacizumab regimen. Major limitations of this study include its retrospective nature, small sample size, and the relatively short follow-up time. Additionally, the heterogenous sequence and timing of immunotherapy in relation to the Y90-RE treatment limit the interpretation of this study’s results and provide limited conclusions as to what the ‘best timing’ of Y90-RE would be in relation to the start of immunotherapy. 

## 5. Conclusions 

The utilization of Y90-RE concurrently or within 90 days of nivolumab or atezolizumab/bevacizumab immunotherapy was associated with a low incidence of severe adverse events and no mortality among this study’s patients with advanced unresectable HCC. While this study’s reported median OS and tumor response rates are encouraging and comparable to published studies, the safety outcomes reported in this study should further promote the prospective studies that are currently ongoing or about to be started. The future of HCC treatment, particularly that involving combined locoregional plus systemic therapies, remains very promising.

## Figures and Tables

**Table 1:** Baseline demographic and clinical characteristics.

Characteristic	Total (N = 19)	Atezolizumab + Bevacizumab (N = 10)	Nivolumab (N = 9)	*p*-Value
Median age (IQR), year	69 (64–73)	66 (62–70)	72 (67–76)	0.2
Male gender, no. (%)	16 (84)	10 (100)	6 (67)	0.02 *
Ethnicity, no. (%)				
Caucasian	11 (58)	8 (80)	3 (34)	0.06
African American	6 (32)	2 (20)	4 (44)	
Asian	2 (10)	0 (0)	2 (22)	
Present etiologies of liver disease, no. (%)				
Hepatitis B virus	1 (5)	0 (0)	1 (11)	0.2
Hepatitis C virus	9 (53)	7 (70)	2 (22)	0.09
EtOH abuse	4 (21)	2 (20)	2 (22)	0.9
Non-alcoholic steatohepatitis	2 (11)	0 (0)	2 (22)	0.07
Idiopathic	5 (26)	1 (10)	4 (44)	0.1
ECOG performance status, no. (%)				
0	7 (37)	6 (60)	1 (11)	0.02 *
1	9 (47)	4 (40)	5 (56)	
2	3 (16)	0 (0)	3 (33)	
ALBI grade, no. (%)				
1	9 (47)	5 (50)	4 (44)	0.8
2	10 (53)	5 (50)	5 (56)	
Child–Pugh class, no. (%)				
A	17 (89)	9 (90)	8 (89)	0.9
B	2 (11)	1 (10)	1 (11)	
BCLC stage, no. (%)				
A	1 (5)	1 (10)	0 (0)	0.5
B	2 (11)	1 (10)	1 (11)	
C	16 (84)	8 (80)	8 (89)	
Median MELD (IQR)	10 (7–13)	10 (8–14)	12 (7–15)	0.6
Median AAPR (IQR)	0.37 (0.29–0.50)	0.35 (0.28–0.51)	0.38 (0.26–0.53)	0.7
Alpha-fetoprotein ≥ 400 ng/mL, no. (%)	7 (37)	5 (50)	2 (22)	0.2
Prior local therapy to targeted lesion(s), no. (%)	5 (26)	3 (30)	2 (22)	0.7
Prior systemic therapy, no. (%)	3 (16)	0 (0)	3 (33)	0.2
Median months since diagnosis to protocol start date (IQR)	11.5 (1.7–33.6)	10.6 (1.7–34.2)	11.5 (5.3–28.7)	0.7

* = Significant *p*-value.

**Table 2:** Baseline imaging characteristics.

Characteristic	Total (N = 19)	Atezolizumab + Bevacizumab (N = 10)	Nivolumab (N = 9)	*p*-Value
Received Y90-RS, no. (%)	7 (37)	4 (40)	3 (33)	0.8
Received resin-based Y90-RE, no. (%)	8 (42)	4 (40)	4 (44)	0.8
Cirrhosis, no. (%)	16 (79)	8 (80)	8 (78)	0.9
Vascular invasion, no. (%)	6 (32)	4 (40)	2 (22)	0.4
Extrahepatic spread, no. (%)	5 (26)	4 (40)	1 (11)	0.1
Presence of bilobar disease, no. (%)	7 (33)	4 (40)	3 (33)	0.8
Presence of non-targeted tumors, no. (%)	7 (37)	3 (30)	4 (44)	0.5
Median targeted tumor size, mm (IQR)				
Largest tumor size	35 (17–68)	36 (19–64)	34 (17–76)	0.9
Cumulative size of tumors	51 (20–83)	51 (26–81)	51 (17–85)	0.9
Median lung shunt fraction (IQR)	5 (4.0–7.7)	4.2 (3.8–5.6)	6.8 (5.4–7.8)	0.1

**Table 3:** Incidences of tumor response, as per the modified Response Evaluation Criteria in Solid Tumors.

Variable	Total (N = 19)	Atezolizumab + Bevacizumab (N = 10)	Nivolumab (N = 9)	*p*-Value
Complete response, no. (%)	3 (16)	1 (10)	2 (22)	0.7
Partial response, no. (%)	8 (42)	5 (50)	3 (33)	
Stable disease, no. (%)	1 (5)	0 (0)	1 (11)	
Progressive disease, no. (%)	2 (11)	1 (10)	1 (11)	
Data missing, no. (%)	5 (26)	3 (30)	2 (22)	
Objective response, no. (%)	11 (58)	6 (60)	5 (56)	0.7
Disease control, no. (%)	12 (63)	6 (60)	6 (67)	0.9

Objective response was defined as complete and/or partial tumor response. Disease control was de-fined as objective response or stable disease.

**Table 4:** Adverse events status post-ablative yttrium-90 transarterial radioembolization, per Common Terminology Criteria for Adverse Events version 5.0.

Characteristic	Total Any Grade (N = 19)	Atezolizumab + Bevacizumab (N = 10)			Nivolumab (N = 9)			Difference in Any Grade Adverse Event (*p*-Value)
Clinical Adverse Events, no. (%)		Any Grade	Grade 1/2	Grade 3	Any Grade	Grade 1/2	Grade 3	
Encephalopathy	0 (0)	0 (0)	0 (0)	0 (0)	0 (0)	0 (0)	0 (0)	0.5
Ascites	4 (21)	3 (30)	3 (30)	0 (0)	1 (11)	1 (11)	0 (0)	0.4
Fatigue	9 (47)	4 (40)	4 (40)	0 (0)	5 (56)	4 (44)	1 (11)	0.6
Abdominal pain	5 (26)	2 (20)	2 (20)	0 (0)	3 (33)	3 (33)	0 (0)	0.2
Nausea	1 (5)	0 (0)	0 (0)	0 (0)	1 (11)	1 (11)	0 (0)	0.3
Vomiting	0 (0)	0 (0)	0 (0)	0 (0)	0 (0)	0 (0)	0 (0)	0.5
Anorexia	1 (5)	1 (10)	1 (10)	0 (0)	0 (0)	0 (0)	0 (0)	0.4
Constipation	0 (0)	0 (0)	0 (0)	0 (0)	0 (0)	0 (0)	0 (0)	0.5
Fever	0 (0)	0 (0)	0 (0)	0 (0)	0 (0)	0 (0)	0 (0)	0.5
Any clinical adverse event	11 (58)	6 (60)	---	---	5 (56)	---	---	0.7
Missing data	3 (16)	1 (10)	---	---	2 (22)	---	---	
Laboratory Adverse Events, No. (%)								
INR	6 (32)	3 (30)	3 (30)	0 (0)	3 (33)	3 (33)	0 (0)	0.6
Aspartate transferase	10 (53)	6 (60)	6 (60)	0 (0)	4 (44)	4 (44)	0 (0)	0.7
Alkaline phosphatase	8 (42)	6 (60)	6 (60)	0 (0)	2 (22)	2 (22)	0 (0)	0.3
Alanine aminotransferase	2 (11)	1 (10)	1 (10)	0 (0)	1 (11)	1 (11)	0 (0)	0.8
Total bilirubin	7 (37)	6 (60)	6 (60)	0 (0)	1 (11)	1 (11)	0 (0)	0.1
Creatinine	4 (21)	2 (20)	2 (20)	0 (0)	2 (22)	2 (22)	0 (0)	0.9
Albumin	8 (42)	5 (50)	5 (50)	0 (0)	3 (33)	3 (33)	0 (0)	0.7
Sodium	12 (63)	6 (60)	6 (60)	0 (0)	6 (67)	6 (67)	0 (0)	0.5
Neutrophils	5 (26)	3 (30)	3 (30)	0 (0)	2 (22)	2 (22)	0 (0)	0.7
Lymphocytes	16 (84)	9 (90)	4 (40)	5 (50)	7 (78)	5 (56)	2 (22)	0.4
White blood count	6 (32)	3 (30)	3 (30)	0 (0)	3 (33)	3 (33)	0 (0)	0.2
Any laboratory adverse event	16 (84)	9 (90)	---	---	7 (78)	---	---	0.5
Missing data	3 (16)	1 (10)	---	---	2 (22)	---	---	

## Data Availability

The data presented in this study can be made available, on request, by the corresponding author. The data is not publicly available due to HIPPA.

## Supplementary Materials

The following supporting information can be downloaded at: https://www.mdpi.com/article/10.3390/curroncol30120734/s1, Table SA: mRECIST Imaging Outcomes at 1 Month; Table SB: Adverse Events at 1 Month, per Common Terminology Criteria for Adverse Events Version 5.0
