# Supplementary material for: Safety and Efficacy of Concurrent Atezolizumab/Bevacizumab or Nivolumab Combination Therapy with Yttrium-90 Radioembolization of Advanced Unresectable Hepatocellular Carcinoma
Source: Curr Oncol. 2023 Nov 25;30(12):10100–10. doi: 10.3390/curroncol30120734 (PMC10742675; doi:10.3390/curroncol30120734)
Supplement: Supplementary file 1 [file curroncol-30-00734-s001.zip › curroncol-2668910-supplementary.pdf]

Supplementary Materials:

Supplementary Table SA - mRECIST Imaging Outcomes at 1 Month

Variable	Total (N=19)	Atezolizumab + Bevacizumab (N=10)	Nivolumab (N=9)	P-value
Complete response, no. (%)	2 (11)	1 (10)	1 (11)	0.5
Partial response, no. (%)	9 (47)	5 (50)	4 (44)	
Stable disease, no. (%)	3 (16)	2 (20)	1 (11)	
Progressive disease, no. (%)	2 (11)	0 (0)	2 (22)	
Data Missing, no. (%)	3 (16)	2 (20)	1 (11)	
Objective response, no. (%)	11 (58)	6 (60)	5 (56)	0.7
Disease control, no. (%)	14 (74)	8 (80)	6 (67)	0.2

Supplementary Table SB - Adverse Events at 1 Month, per Common Terminology Criteria for Adverse Events Version 5.0

**Characteristic**	**Total Any Grade (N=19)**	**Atezolizumab + Bevacizumab (N=10)**			**Nivolumab (N=9)**			**Difference in any grade adverse event (P-value)**
**Clinical Adverse Events, no. (%)**		**Any Grade**	**Grade 1/2**	**Grade 3**	**Any Grade**	**Grade 1/2**	**Grade 3**	
Encephalopathy	0 (0)	0 (0)	0 (0)	0 (0)	0 (0)	0 (0)	0 (0)	0.2
Ascites	5 (26)	2 (20)	2 (20)	0 (0)	3 (33)	3 (33)	0 (0)	0.4
Fatigue	8 (42)	2 (20)	2 (20)	0 (0)	6 (67)	5 (56)	1 (11)	0.1
Abdominal pain	5 (26)	2 (20)	2 (20)	0 (0)	3 (33)	3 (33)	0 (0)	0.4
Nausea	4 (21)	2 (20)	2 (20)	0 (0)	2 (22)	2 (22)	0 (0)	0.5
Vomiting	0 (0)	0 (0)	0 (0)	0 (0)	0 (0)	0 (0)	0 (0)	0.2
Anorexia	2 (11)	0 (0)	0 (0)	0 (0)	2 (22)	2 (22)	0 (0)	0.1
Constipation	1 (5)	0 (0)	0 (0)	0 (0)	1 (11)	1 (11)	0 (0)	0.2
Fever	0 (0)	0 (0)	0 (0)	0 (0)	0 (0)	0 (0)	0 (0)	0.2
Any clinical adverse event	12 (63)	5 (50)	---	---	7 (78)	---	---	0.3
Missing data	1 (5)	1 (10)	---	---	0 (0)	---	---	
**Laboratory Adverse Events, no. (%)**	**Total Any Grade (N=19)**	**Atezolizumab + Bevacizumab (N=10)**			**Nivolumab (N=9)**			**Difference in any grade adverse event (P-value)**
		**Any Grade**	**Grade 1/2**	**Grade 3**	**Any Grade**	**Grade 1/2**	**Grade 3**	
INR	5 (26)	3 (30)	2 (20)	1 (10)	2 (22)	2 (22)	0 (0)	0.3
Aspartate transferase	8 (42)	4 (40)	4 (40)	0 (0)	4 (44)	4 (44)	0 (0)	0.4
Alkaline phosphatase	11 (58)	6 (60)	6 (60)	0 (0)	5 (55)	5 (55)	0 (0)	0.3
Alanine aminotransferase	1 (5)	1 (10)	1 (10)	0 (0)	0 (0)	0 (0)	0 (0)	0.2
Total bilirubin	5 (26)	3 (30)	3 (30)	0 (0)	2 (22)	2 (22)	0 (0)	0.3
Creatinine	3 (16)	2 (20)	2 (20)	0 (0)	1 (11)	0 (0)	1 (11)	0.1
Albumin	8 (42)	2 (20)	2 (20)	0 (0)	6 (66)	6 (66)	0 (0)	0.02*

Sodium	11 (58)	6 (60)	6 (60)	0 (0)	5 (55)	3 (33)	2 (22)	0.2
Neutrophils	2 (11)	0 (0)	0 (0)	0 (0)	2 (22)	2 (22)	0 (0)	0.1
Lymphocytes	12 (63)	5 (50)	3 (30)	2 (20)	7 (78)	2 (22)	5 (56)	0.3
White blood count	5 (26)	2 (20)	2 (20)	0 (0)	3 (33)	3 (33)	0 (0)	0.4
Any laboratory adverse event	18 (95)	9 (90)	---	---	9 (100)	---	---	0.2
Missing data	1 (5)	1 (10)	---	---	0 (0)	---	---	
